# Once‐daily glatiramer acetate decreases magnetic resonance imaging disease activity in Japanese patients with relapsing–remitting multiple sclerosis

**DOI:** 10.1111/cen3.12383

**Published:** 2017-03-23

**Authors:** Takashi Yamamura, Natalia Ashtamker, David Ladkani, Toshiyuki Fukazawa, Hideki Houzen, Masami Tanaka, Toshiro Miura, Volker Knappertz

**Affiliations:** ^1^ Department of ImmunologyNational Center of Neurology and PsychiatryNational Institute of Neuroscience, and Multiple Sclerosis CenterTokyoJapan; ^2^ Research and DevelopmentTeva Pharmaceutical IndustriesNetanyaIsrael; ^3^ Sapporo Neurology HospitalSapporoJapan; ^4^ Department of NeurologyObihiro Kosei General HospitalHokkaidoJapan; ^5^ Multiple Sclerosis CenterKyoto Min‐iren Chuo HospitalKyotoJapan; ^6^ Department of NeurologyKaikoukai Josai HospitalNagoyaJapan; ^7^ Department of NeurologySchool of MedicineFujita Health UniversityAichiJapan; ^8^ Research and DevelopmentTeva Pharmaceutical K.K.TokyoJapan; ^9^ Research and DevelopmentTeva Pharmaceutical IndustriesFrazerPAUSA; ^10^ Department of NeurologyHeinrich Heine UniversityDüsseldorfGermany

**Keywords:** gadolinium‐enhancing lesions, glatiramer acetate, Japanese, relapsing–remitting multiple sclerosis, T_2_ lesions

## Abstract

**Objective:**

Multiple sclerosis (MS) prevalence, clinical patterns, and treatment responses vary between races and geographical latitudes. Glatiramer acetate (GA; Copaxone) has provided a safe, effective treatment option for relapsing–remitting MS patients in the USA, European nations, and other countries for decades. The objective of the present study was to assess the safety and efficacy of GA in reducing magnetic resonance imaging disease activity in Japanese patients with active relapsing–remitting MS.

**Methods:**

This phase 2, multicenter, open‐label, single‐arm, 52‐week study measured the effect of GA 20 mg once‐daily on magnetic resonance imaging disease activity. GA efficacy was evaluated through week 36, and safety through week 52. The primary end‐point was change in the mean number of T_1_‐weighted gadolinium‐enhancing (GdE) lesions from pretreatment (weeks –8, –4 and baseline) to weeks 28, 32 and 36. Secondary end‐points included a change in mean number of new T_2_‐weighted lesions, GdE lesion and T_2_ lesion volumes, annualized relapse rate, and Expanded Disability Status Scale scores.

**Results:**

GA therapy reduced the number of new GdE lesions by 65.66% (95% CI 33.19–82.35%). The number of new T_2_ lesions and GdE lesion volume were also reduced from pretreatment. The annualized relapse rate was reduced by 42% compared with the 1 year before treatment. Changes in T_2_ lesion volume and Expanded Disability Status Scale scores were favorable, but less pronounced. Most common adverse events were injection‐site reactions.

**Conclusions:**

The present study confirmed the well‐established safety, tolerability and efficacy profile of GA in Japanese MS patients.

## Introduction

Multiple sclerosis (MS) is an inflammatory, chronic disease of the central nervous system mainly affecting young adults.^1^ The median estimated global incidence and prevalence are 2.5 and 30 per 100 000 persons, respectively.^1^ Estimates vary widely, and are influenced by genetic susceptibility, race, geographic and environmental factors, and the availability of diagnostic tools, including magnetic resonance imaging (MRI) scanners.^1^ Although the prevalence of MS in Japan is relatively low (approximately 14 persons per 100 000) compared with Western countries, several studies show that it is increasing.^2^

Axonal injury can occur early in MS, and is associated with progressive and permanent disability.^3^ Most persons with MS are initially diagnosed with relapsing–remitting disease (RRMS), which is characterized by periods of symptomatic relapse followed by periods of remission. There is no cure for MS, and treatment is focused on reducing the relapse rate, controlling symptoms and delaying disease progression. Early intervention with disease‐modifying therapies can reduce or prevent accumulating neurodegeneration.^4^

Glatiramer acetate (GA; Copaxone, Teva Pharmaceutical Industries, Petach Tikva, Israel) is a synthetic mixture of polypeptide chains produced from four amino acids in a fixed ratio.^5^, ^6^ In clinical trials, GA significantly reduced disease activity, as evidenced by fewer GdE lesions^7^ and lower annualized relapse rates (ARR) in patients with RRMS.^8^, ^9^ GA is safe and well‐tolerated, and its effects on disability and relapse are sustained with continuous use.^10^, ^11^ In patients presenting with clinically isolated syndrome and brain MRI lesions, early treatment with GA delayed both a second attack and conversion to clinically definite MS, and reduced MRI activity.^12^

GA is indicated as a first‐line disease‐modifying therapy for patients with RRMS, and is approved in 57 countries.^5^ In September 2015, GA was approved in Japan for treatment of RRMS based on the results of three pivotal clinical trials that led to approvals in other countries, and based on the safety and efficacy results of a phase 2 trial carried out in Japanese patients.^7^, ^8^, ^12^ The purpose of the present study was to assess the safety, tolerability, and efficacy of GA on MRI measures of disease activity and immune responses in Japanese patients with RRMS.

## Methods

The present multicenter, open‐label, single‐arm study of GA in Japanese patients with RRMS was carried out in accordance with the Declaration of Helsinki and Good Clinical Practice guidelines, and was approved by the institutional review board of each study site. All patients provided written informed consent before participating.

### Patients

Japanese patients aged 18–60 years with confirmed RRMS (2005 Revised McDonald Criteria^13^) were eligible. Patients were required to have experienced ≥1 documented relapse within the year before the screening visit (week –8), but be relapse‐free for ≥30 days before screening, and to have one to 15 GdE lesions on any of three screening MRI scans. Eligibility was limited to patients with an Expanded Disability Status Scale (EDSS) score of 0–5.^14^

Exclusion criteria included use of any of the following before screening: corticosteroids or adrenocorticotropic hormones (within 30 days), immunosuppressive treatments (within 6 months), azathioprine (within 90 days), interferon‐β (within 60 days) or intravenous (i.v.) immunoglobulins (within 60 days). Any previous use of cladribine, natalizumab, total body or lymphoid irradiation, or stem cell treatment was also disallowed, as were the use of plasma exchange or lymphocytapheresis therapy within 6 months of screening or chronic (>30 consecutive days) corticosteroid treatment (within 60 days). Short‐term therapy with methylprednisolone for investigator‐confirmed relapses was permissible.

Patients with a diagnosis of opticospinal MS and those who had previously used GA were excluded. Women were required to practice an acceptable form of birth control.

### Study schedule

After an 8‐week pretreatment screening phase, GA 20 mg/mL was administered subcutaneously (SC) by self‐injection once‐daily for 52 weeks. Phase 1, the efficacy phase of the study, included the 44‐week period between the start of the screening phase and the end of the first 36 weeks of treatment. Phase 2 began at week 36 and ended at week 52 (study end). Efficacy and safety measures were evaluated up to week 36 and week 52, respectively.

### End‐points

The primary end‐point was reduction in the total number of GdE lesions during weeks –8, –4, and 0 compared with post‐treatment (weeks 28, 32 and 36). MRI scans carried out within 30 days after i.v. corticosteroid administration were excluded from analyses. Secondary end‐points included the number of GdE lesions at each specified time‐point, number of new T_2_ lesions (measured by the change in the total number of new T_2_ lesions from pretreatment to weeks 32 and 36), and volumes of GdE and T_2_ lesions (measured by the change in both from baseline to each specified time‐point). Pretreatment and post‐treatment ARR, and changes in EDSS, functional systems and ambulation index scores were also assessed.

Safety was assessed by adverse events (AE), laboratory tests, vital signs, electrocardiogram and physical examinations. Proliferative responses of peripheral blood mononuclear cells, presence of anti‐GA antibodies and anti‐aquaporin (AQP) 4 antibody (measured at week –8 for reference) were analyzed as immunological safety end‐points. AE were coded using MedDRA/J Version 17 (MedDRA MSSO, McLean, VA, USA).

### Statistical analysis

Assuming that the change in the total number of GdE lesions from pretreatment to weeks 28, 32 and 36 would be ≥25%, at least 50 participants would be required to obtain a one‐sided α error rate of 2.5% with a statistical power of 80% using a repeated measures negative binomial regression model.

Three datasets were defined: (i) the full analysis population included all patients who received at least one dose of GA and had at least one MRI in weeks 28, 32 or 36/early termination discontinuation; (ii) the per‐protocol population included patients who completed phase 1 without significant protocol deviations; and (iii) the safety analysis set included all patients who received at least one dose of GA.

The primary analysis evaluated the change in the total number of GdE lesions between pretreatment and the post‐treatment periods for patients in the full analysis set, using a negative binomial regression model (PROC GENMOD of SAS select DIST = NEGBIN as MODEL option; SAS Institute, Cary, NC, USA) with time, age at screening and disease duration as covariates. The primary efficacy end‐point was met if the lower limit of the 95% confidence interval (CI) of the change in the total number of GdE lesions from pretreatment to the last 3 months of the first treatment period was ≥25%. Missing MRI data were corrected using an “offset” method based on the logarithm of the percentage of available scans relative to the maximum number of scans for that time‐period.

Two preplanned sensitivity analyses of the primary efficacy end‐point were carried out by repeating the primary analysis: (i) without the covariates; and (ii) by Wilcoxon's signed‐rank test. Three additional post‐hoc sensitivity analyses were also carried out. They included non‐parametric and parametric analyses of all MRI data available regardless of whether a patient discontinued before week 36 or whether the scans were taken within 30 days of receipt of i.v. corticosteroids; and a parametric analysis of MRI data only for patients who completed 36 weeks of GA treatment and excluded MRI scans taken within 30 days of i.v. corticosteroid use.

Analyses similar to the primary analysis evaluated the change in total number of new T_2_ lesions from pretreatment (weeks –4 and baseline) to weeks 32 and 36. Over‐dispersed Poisson regression using quasi‐likelihood estimation (SAS PROC GENMOD; SAS Institute) was used to estimate the change in relapse rate from pre‐ to post‐treatment, and the ARR during the treatment period with corresponding 90% CI. Summary descriptive statistics were calculated for EDSS, functional systems and ambulation index scores, and for changes in these scores from baseline to each specified time‐point.

## Results

### Study population

**Figure 1:**
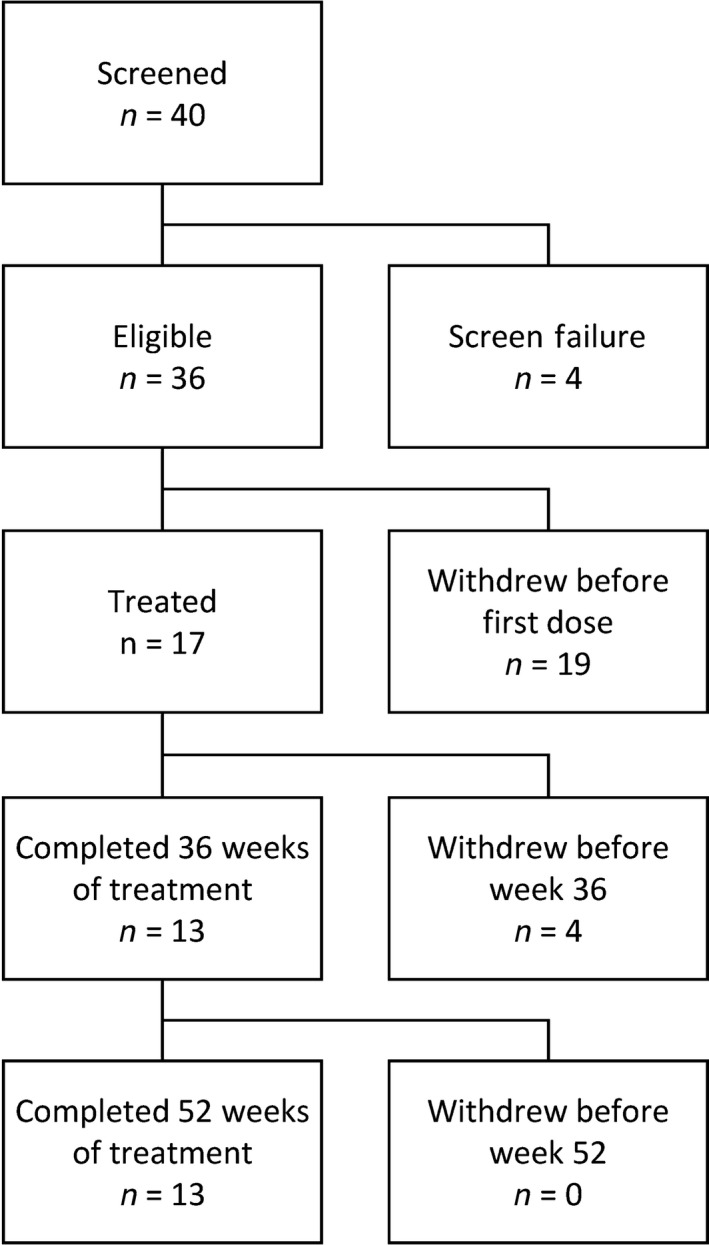
Patient deposition. Of the 36 eligible patients, 19 withdrew before treatment: four due to the judgment of a principal investigator or subinvestigator, and 15 due to violating exclusion criteria or deviating from inclusion criteria.

The present study was carried out at eight sites in Japan. Recruitment was unexpectedly slow; more than 1 year after study start, just 17 patients had enrolled and received the study drug. The protocol was amended before analyses of results to limit the study to these 17 patients. Four patients withdrew before week 36 (three of whom withdrew before week 28 and had no post‐treatment MRI scan for efficacy assessment); three because of an AE (aggravation of MS, injection site pain and injection site erythema) and one due to investigator discretion because of MS relapse. Mean (SD) duration of GA exposure was 301.8 days (125.7 days). The median rate of compliance with GA treatment from baseline to week 52/termination was 99.7% (range 53.6–100.0%). Patient disposition is shown in Figure 1.

Most patients in the study were women (94.1%), ages ranged from 29 to 55 years and the mean disease duration was approximately 7 years (Table 1). On average, patients experienced two relapses within the 1‐year period before study enrollment (range 1–10), and the total number of GdE lesions at baseline recorded during the screening visits ranged from 1 to 27.

**Table 1:** Demographic and baseline multiple sclerosis clinical characteristics (week 0)

Parameter	*n* (%)
Sex, *n* (%)	
Men	1 (5.9)
Women	16 (94.1)
Mean age, years [SD]	38.8 [7.5]
Mean disease duration, months [SD]	84.79 [83.74]
Mean EDSS score [SD]	2.47 [1.22]
Mean no. relapses in prior year [SD]	2.0 [2.2]
No. GdE lesions [SD]	6.6 [7.8]
No. new T_2_ lesions^a^ [SD]	4.2 [5.7]

EDSS, Expanded Disability Status Scale; GdE, gadolinium‐enhancing; SD, standard deviation.

a Three magnetic resonance imaging scans were carried out in the pretreatment period; at week –8 (screening magnetic resonance imaging where the total number of T_2_ lesions was assessed), week –4 (new T_2_ lesions were assessed) and week 0 (baseline, new T_2_ lesions were assessed).

Full analysis set included 17 treated patients with multiple sclerosis.

### Efficacy

The full analysis population included 17 patients who received ≥1 dose of GA and had at least one MRI examination at week 28, 32 or 36/early discontinuation. The per‐protocol population comprised 13 patients who had completed study phase 1 (i.e. week 36).

### Primary end‐point

**Figure 2:**
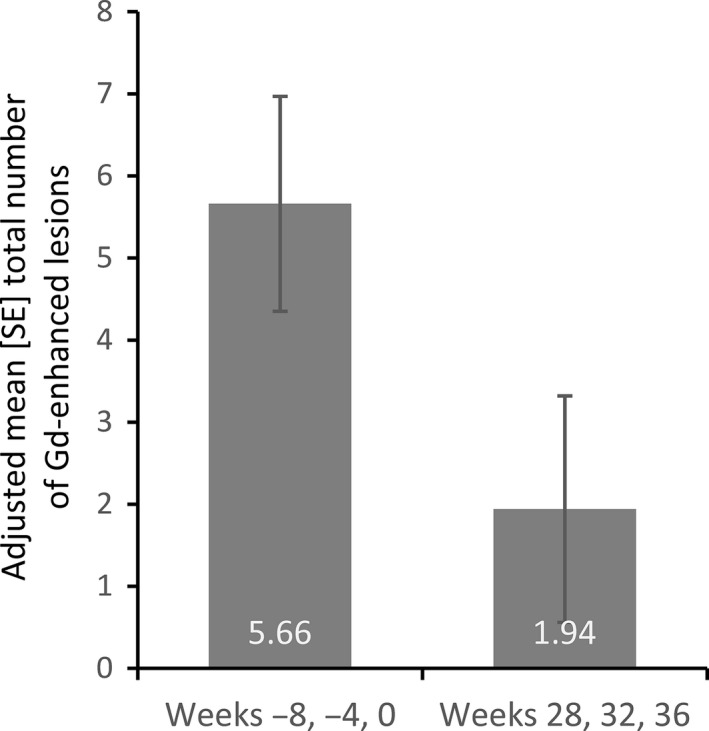
Effect of glatiramer acetate on the total number of gadolinium (Gd)‐enhancing lesions (standard error). The sum of Gd‐enhancing lesions at pretreatment (*n* = 17) was compared with the sum of Gd‐enhancing lesions post‐treatment (*n* = 14).

Although the study was underpowered, the primary end‐point was met; there was a significant reduction in the total number (SE) of GdE lesions from an adjusted mean of 5.66 (1.31) lesions pretreatment to an adjusted mean of 1.94 (1.38) during GA treatment (Fig. 2), reflecting a reduction of 65.66% (95% CI 33.19–82.35). The lower limit of the 95% CI (33.19%) exceeded the predefined 25% threshold, showing a significant reduction in MRI disease activity. Similar results were observed for patients in the per‐protocol data set (72.07% reduction in GdE lesions; 95% CI 49.50–84.55).

In the preplanned sensitivity analysis using the negative binominal regression method without adjustment for covariates, a significant decrease in the total number of GdE lesions with GA treatment was observed (65.18%, 95% CI 30.82–82.47). The second preplanned sensitivity analysis using Wilcoxon's signed rank test also showed a significant reduction in the total number of GdE lesions post‐GA treatment (*n* = 14; *P* = 0.0176), consistent with the primary analysis.

Results of the post‐hoc parametric sensitivity analysis, which included only patients (*n* = 14) who had not terminated before week 28 and MRI scans taken within 30 days of i.v. corticosteroid use, supported the primary analysis; the total number of GdE lesions decreased from 4.64 pretreatment to 1.74 post‐treatment (*P* = 0.0176), showing a change rate of 62.5% (95% CI 24.03–81.49). In the post‐hoc non‐parametric sensitivity analysis that included MRI scans from all 17 patients and scans taken within 30 days of i.v. corticosteroid use, the difference between pre‐ and post‐GA treatment in GdE lesions yielded a borderline significant result (*P* = 0.0682). The post‐hoc parametric sensitivity analysis using the same parameters showed minimal effect on the change from pre‐ to post‐treatment number of GdE lesions (−2.31%), and the lower limit of the 95% CI was below the 25% threshold.

### Secondary end‐points

**Figure 3:**
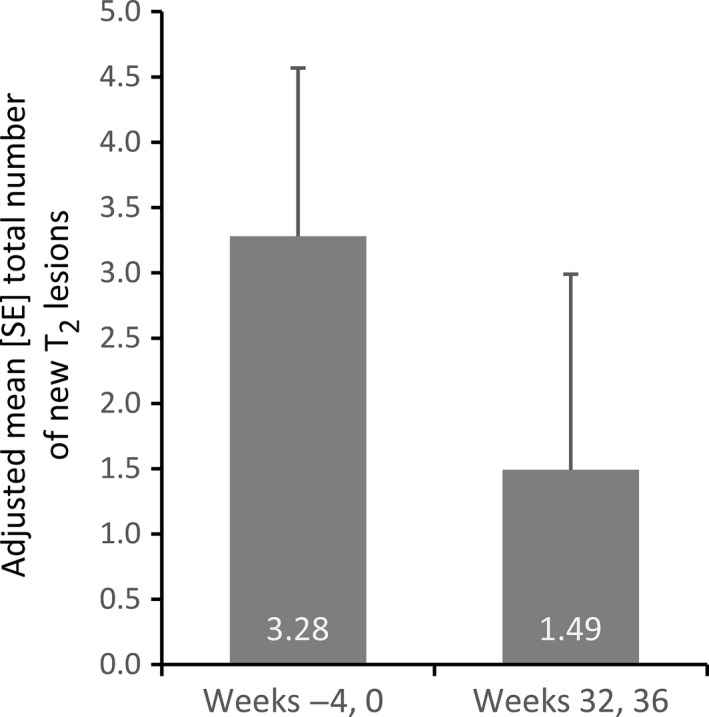
Effect of glatiramer acetate on adjusted mean (standard error) total number of new T_2_ lesions. The full analysis set consisted of 17 patients. The sum of the new T_2_‐weighted lesions at weeks –4 and 0 (*n* = 17) was compared with the sum at weeks 32 and 36 post‐treatment (*n* = 14).

MRI secondary end‐points for the full analysis population showed decreases from pretreatment to post‐treatment time‐points. Mean (SD) total number of pretreatment GdE lesions ranged from 1.6 (1.4) in week –8 to 2.8 (3.6) in week 0, then decreased during treatment to 1.1 (1.4) in week 12, 1.0 (2.1) in week 28, 0.8 (1.3) in week 32 and 1.4 (3.7) in week 36. The mean change in GdE lesion volume from baseline was negative, reflecting an effect in favor of GA at all post‐treatment measurements (−0.200 mL at week 28, −0.187 mL at week 32 and −0.127 mL at week 36). The point estimate of the total number of new T_2_ lesions decreased from an adjusted pretreatment mean of 3.28 (weeks –4 and 0) to 1.49 post‐treatment (weeks 32 and 36), reflecting a reduction in favor of GA by 54.36% (90% CI 12.81–76.12; Fig. 3), supporting the effect obtained in reducing GdE lesions. T_2_ lesion volume also decreased.

Similar to the favorable effect on MRI measures, there were signs of improvements in clinical end‐points. ARR was reduced for all 17 patients by 42% (90% CI 6.50–63.94), from an adjusted mean of 1.84 relapses/year before treatment to 1.07 relapses/year during treatment. EDSS scores remained unchanged for most patients or showed small decreases (mean [SD] change −0.25 [0.75] at week 36).

### Safety and tolerability

Safety was evaluated for all 17 patients. In all, 93% of AE were classified as potentially related to the study drug. All 17 patients experienced at least one AE during the study. AE reported in ≥3 patients are shown in Table 2. Most AE considered to be drug‐related involved local injection site reactions (e.g. erythema, pain, induration).

**Table 2:** Most common adverse events occurring in ≥3 patients

Preferred term	All patients (*n* = 17)		
	No. patients	Incidence (%)	No. events
Injection site erythema	15	88.2	221
Injection site pain	15	88.2	87
Injection site induration	13	76.5	118
Injection site pruritus	12	70.6	107
Injection site swelling	11	64.7	125
Nasopharyngitis	8	47.1	10
Injection site irritation	7	41.2	97
Injection site bruising	7	41.2	15
Injection site warmth	6	35.3	29
Pyrexia	4	23.5	8
Malaise	3	17.6	5
Palpitations	3	17.6	15

Four serious AE were reported in three patients (18%): MS was reported as a serious AE in two patients, and both events were considered by the investigator to be related to the study drug; MS relapse was reported for one patient, which was also considered to be drug‐related; and sinus bradycardia was reported for one patient, which was not considered to be related to GA treatment. There were no deaths. All AE were rated as mild or moderate severity. The only moderate AE reported in ≥3 patients was injection site erythema (*n* = 4; 24%). Four drug‐related AE in four patients led to study discontinuation (MS relapse, MS, injection‐site swelling and abnormal brain MRI). All AE but the abnormal brain MRI resolved by the end of follow up.

### Chemistry, immunological and laboratory evaluations

Mild biochemical and hematological changes were noted in a few patients; none were serious or led to discontinuation of GA. Changes in urinalysis, vital signs, electrocardiogram and bodyweight were non‐significant. Peripheral blood mononuclear cells from seven patients with available data showed no changes in proliferative response during GA treatment. Sera for all patients were positive for anti‐GA reactive immunoglobulin G antibodies on at least one time‐point during treatment. Tests for anti‐AQP4 antibodies carried out at screening (week –8) were negative in all 17 patients.

## Discussion

The intention of the present study was to enroll 50 patients from 17 Japanese sites. Perhaps because of the low prevalence of MS in Japan,^1^ recruitment was slow, with just 17 patients enrolled at eight sites more than 1 year after study initiation. The prevalence of MS in Japan contrasts sharply with that of Western regions, such as Europe, where the prevalence has been reported to be as high as 80/100 000.^1^ Among Asian populations, MS is considered to have more frequent involvement of the optic nerve and spinal cord, fewer brain lesions, longer spinal cord lesions, a higher female‐to‐male ratio, older age of onset, more rapid progression of disability, and a less frequent incidence of a progressive course.^2^, ^15^, ^16^, ^17^ Prevalence and disease differences between Caucasians and Asians could reflect distinct genetic, metabolic, and lifestyle differences between these ethnic groups.^18^ For example, the most common form of MS in Japanese patients is a subtype associated with the HLA‐DRB1*0405 allele, which is found in more than 40% of all Japanese MS patients; whereas, the *DR15* haplotype and its individual alleles are more strongly associated with MS risk in Caucasians.^2^ Carriers of the HLA‐DRB1*0405 allele who do not have neuromyelitis optica (NMO) spectrum disorders show fewer brain lesions on MRI and have a relatively benign disease course, which might also have hindered patient accrual if many Japanese patients could not meet study eligibility criteria related to relapse or MRI activity.^2^ Reported differences between Asian and Western MS populations might also be due to the fact that a significant proportion of patients with NMO/NMO spectrum disorders, which are more prevalent in Asian patients, might have been included in previous Asian MS trials.^17^ NMO can be distinguished with 76% sensitivity by AQP4 antibody seropositivity.^19^ In the current study, all participants were negative for anti‐AQP4 antibodies at screening (week –8), suggesting that patients with NMO were not enrolled into the study.

Despite the small number of patients in the study, GA resulted in a statistically significant and clinically meaningful reduction of MS disease activity on MRI as measured by a greater reduction in the total number of GdE lesions, an indicator of active disease, than the predefined threshold in these RRMS patients in Japan. These findings were confirmed using multiple sensitivity analyses of the primary end‐point. Safety and tolerability outcomes were also comparable with those of previous studies in Caucasian patients, with no new safety concerns emerging.^7^, ^8^

These findings in Japanese patients are consistent with those reported by Comi et al.^12^ for a larger RRMS patient cohort treated with GA (*n* = 119), which showed a significant reduction in total GdE lesions over 36 weeks with GA versus a placebo in mainly Caucasian patients. In the current study, favorable reductions during GA treatment were also observed for secondary end‐points, including the number of new T_2_ lesions and GdE lesion volume, in patients who completed the initial 36‐week treatment phase. For all patients, ARR was substantially reduced by ~42% compared with ARR in the 1 year before GA treatment.

One limitation of these data was the small number of participating patients. The patient sample size in the present study could increase the risk of both type 1 and 2 errors; however, results of predefined and post‐hoc sensitivity analyses that included early discontinuation scans showed the robustness of the GA treatment effect on GdE lesions. The single‐arm, open‐label design has well understood limitations on distinguishing treatment effects from regression to the mean effects. Regression to the mean effect occurs in activity‐enriched patient populations with MS, whereby MRI and clinical activity attenuates over the first 0–6 months after randomization.^20^ However, reductions in GdE lesions in the current study were not very different from those reported in the GALA study of GA 40 mg administered thrice‐weekly, which had a placebo control (44.8% reduction in GdE lesions *vs* placebo).^21^ Furthermore, a meta‐analysis of 21 phase 2 and phase 3 MS trials showed that the number of GdE lesions at baseline was a good predictor of short‐term MRI disease activity, specifically, the number of GdE lesions after 6 months of treatment, and might be used to control for regression to the mean effects in non‐placebo‐controlled studies.^20^

Another potential weakness was the gender imbalance in study participants (16/17 participants were female). The female‐to‐male ratio of MS patients in Japan is approximately three to one.^2^ Participation of a single male patient limits the generalizability of results to Japanese men with RRMS; however, it is reassuring that there has been no evidence in clinical data reported to date to suggest a gender bias in response to GA by RRMS patients.^22^, ^23^

Safety results in this Japanese population were consistent with those reported for GA 20 mg/day in predominantly Caucasian patients.^7^, ^8^, ^24^ Injection site reactions were the predominant AE. With the exception of one case of sinus bradycardia, serious AE were related to underlying disease. Proliferation of peripheral blood mononuclear cells was unchanged during GA treatment; evidence shows that GA induces an anti‐inflammatory type II cytokine milieu in antigen presenting cells, T cells, and B cells *in vitro* and *in vivo*.^25^, ^26^, ^27^, ^28^, ^29^ Because the development of neutralizing antibodies can interfere with biological response to some disease‐modifying therapies (notably interferon‐β^30^) serum samples were tested for the presence of anti‐GA antibodies. GA works as an antigen‐based therapeutic vaccine, and as expected, sera of all patients were positive for anti‐GA reactive immunoglobulin G antibodies in at least one tested time‐point.^31^ These antibodies have no effect on the efficacy or safety of GA.^32^

In summary, GA was associated with significant improvement in GdE MRI lesions, and the safety and efficacy of GA in these Japanese patients were consistent with those in Western Caucasian populations.

## Conflict of interest

Dr Takashi Yamamura has served on scientific advisory boards for Biogen Idec and Takeda Pharmaceutical; has received travel funding and/or speaker honoraria from Biogen Idec, Dainippon Sumitomo Pharma, Bayer Holding, Chugai Pharmaceutical, Takeda Pharmaceutical, Ono Pharmaceutical and Mitsubishi Tanabe Pharma; and has received research support from Chugai Pharmaceutical, Teva Pharmaceutical K.K., Takeda Pharmaceutical, Novartis Pharma, Nihon Pharmaceutical, Biogen Idec and Asahi Kasei Kuraray Medical. Dr Natalia Ashtamker, Dr David Ladkani, Dr Toshiro Miura and Dr Volker Knappertz are employees of, and hold stock/equity in, Teva Pharmaceutical Industries. Dr Toshiyuki Fukazawa serves/has served on scientific advisory boards for Bayer Pharma, Biogen Idec, Mitsubishi Tanabe Pharma, Takeda Pharmaceutical and Novartis Pharma; and has received funding for travel and speaker honoraria from Bayer Pharma, Biogen Idec, Mitsubishi Tanabe Pharma, Takeda Pharmaceutical and Novartis Pharma. Dr Hideki Houzen serves on the scientific advisory board for Biogen Idec and Mitsubishi Tanabe Pharma, and has received speaker honoraria from Takeda Pharmaceutical. Dr Masami Tanaka received speaker honoraria from Biogen Idec Japan, Bayer Schering Pharma, Asahi Kasei Medical, Novartis Pharma, Takeda Pharmaceutical and Mitsubishi Tanabe Pharma. The research sites of Dr Yamamura, Dr Fukazawa, Dr Houzen and Dr Tanaka received research support from Teva Pharmaceutical K.K. for the study of this report, and from Takeda Pharmaceutical for the follow‐up study from this report.

## References

[1] World Health Association . Atlas: Multiple Sclerosis Resources in the World 2008. (accessed March 2015).

[2] Kira J‐i . Genetic and environmental factors underlying the rapid changes in epidemiological and clinical features of multiple sclerosis and neuromyelitis optica in Japanese. Clin Exp Neuroimmunol. 2013; 4: 261–73.

[3] De Stefano N , Narayanan S , Francis GS , et al. Evidence of axonal damage in the early stages of multiple sclerosis and its relevance to disability. Arch Neurol. 2001; 58: 65–70. 11176938 10.1001/archneur.58.1.65

[4] Rizvi SA , Kim E , Moodie J . Glatiramer in the treatment of multiple sclerosis. Int J Nanomedicine. 2006; 1: 283–9. 17717969 PMC2426806

[5] Weinstock‐Guttman B . An update on new and emerging therapies for relapsing‐remitting multiple sclerosis. Am J Manag Care. 2013; 19: s343–54. 24494635

[6] Garg N , Smith TW . An update on immunopathogenesis, diagnosis, and treatment of multiple sclerosis. Brain Behav. 2015; 5: e00362. 26445701 10.1002/brb3.362 PMC4589809

[7] Comi G , Filippi M , Wolinsky JS . European/Canadian multicenter, double‐blind, randomized, placebo‐controlled study of the effects of glatiramer acetate on magnetic resonance imaging–measured disease activity and burden in patients with relapsing multiple sclerosis. European/Canadian Glatiramer Acetate Study Group. Ann Neurol. 2001; 49: 290–7. 11261502

[8] Johnson KP , Brooks BR , Cohen JA , et al. Copolymer 1 reduces relapse rate and improves disability in relapsing‐remitting multiple sclerosis: results of a phase III multicenter, double‐blind placebo‐controlled trial. The Copolymer 1 Multiple Sclerosis Study Group. Neurology. 1995; 45: 1268–76. 7617181 10.1212/wnl.45.7.1268

[9] Ziemssen T , Gilgun‐Sherki Y . Sub‐analysis of geographical variations in the 2‐year observational COPTIMIZE trial of patients with relapsing‐remitting multiple sclerosis converting to glatiramer acetate. BMC Neurol. 2015; 15: 189. 26450155 10.1186/s12883-015-0448-4 PMC4599648

[10] Ford C , Goodman AD , Johnson K , et al. Continuous long‐term immunomodulatory therapy in relapsing multiple sclerosis: results from the 15‐year analysis of the US prospective open‐label study of glatiramer acetate. Mult Scler. 2010; 16: 342–50. 20106943 10.1177/1352458509358088 PMC2850588

[11] Boster AL , Ford CC , Neudorfer O , Gilgun‐Sherki Y . Glatiramer acetate: long‐term safety and efficacy in relapsing‐remitting multiple sclerosis. Expert Rev Neurother. 2015; 15: 575–86. 25924547 10.1586/14737175.2015.1040768

[12] Comi G , Martinelli V , Rodegher M , et al. Effect of glatiramer acetate on conversion to clinically definite multiple sclerosis in patients with clinically isolated syndrome (PreCISe study): a randomised, double‐blind, placebo‐controlled trial. Lancet. 2009; 374: 1503–11. 19815268 10.1016/S0140-6736(09)61259-9

[13] Polman CH , Reingold SC , Edan G , et al. Diagnostic criteria for multiple sclerosis: 2005 revisions to the “McDonald Criteria”. Ann Neurol. 2005; 58: 840–6. 16283615 10.1002/ana.20703

[14] Kurtzke JF . Rating neurologic impairment in multiple sclerosis: an expanded disability status scale (EDSS). Neurology. 1983; 33: 1444–52. 6685237 10.1212/wnl.33.11.1444

[15] Kira J . Multiple sclerosis in the Japanese population. Lancet Neurol. 2003; 2: 117–27. 12849268 10.1016/s1474-4422(03)00308-9

[16] Kira J . Neuromyelitis optica and asian phenotype of multiple sclerosis. Ann N Y Acad Sci. 2008; 1142: 58–71. 18990121 10.1196/annals.1444.002

[17] Piccolo L , Kumar G , Nakashima I , et al. Multiple sclerosis in Japan appears to be a milder disease compared to the UK. J Neurol. 2015; 262: 831–6. 25605435 10.1007/s00415-015-7637-3

[18] Isobe N , Oksenberg JR . Genetic studies of multiple sclerosis and neuromyelitis optica: current status in European, African American and Asian populations. Clin Exp Neuroimmunol. 2014; 5: 61–8.

[19] Wingerchuk DM , Lennon VA , Pittock SJ , Lucchinetti CF , Weinshenker BG . Revised diagnostic criteria for neuromyelitis optica. Neurology. 2006; 66: 1485–9. 16717206 10.1212/01.wnl.0000216139.44259.74

[20] Stellmann JP , Sturner KH , Young KL , Siemonsen S , Friede T , Heesen C . Regression to the mean and predictors of MRI disease activity in RRMS placebo cohorts–is there a place for baseline‐to‐treatment studies in MS? PLoS One. 2015; 10: e0116559. 25659100 10.1371/journal.pone.0116559 PMC4319835

[21] Khan O , Rieckmann P , Boyko A , Selmaj K , Zivadinov R , Group GS . Three times weekly glatiramer acetate in relapsing‐remitting multiple sclerosis. Ann Neurol. 2013; 73: 705–13. 23686821 10.1002/ana.23938

[22] Fogarty E , Schmitz S , Tubridy N , Walsh C , Barry M . Comparative efficacy of disease‐modifying therapies for patients with relapsing remitting multiple sclerosis: systematic review and network meta‐analysis. Mult Scler Relat Disord. 2016; 9: 23–30. 27645339 10.1016/j.msard.2016.06.001

[23] Martinelli‐Boneschi F , Rovaris M , Johnson KP , et al. Effects of glatiramer acetate on relapse rate and accumulated disability in multiple sclerosis: meta‐analysis of three double‐blind, randomized, placebo‐controlled clinical trials. Mult Scler. 2003; 9: 349–55. 12926839 10.1191/1352458503ms932oa

[24] Johnson KP , Brooks BR , Cohen JA , et al. Extended use of glatiramer acetate (Copaxone) is well tolerated and maintains its clinical effect on multiple sclerosis relapse rate and degree of disability. Copolymer 1 Multiple Sclerosis Study Group. Neurology. 1998; 50: 701–8. 9521260 10.1212/wnl.50.3.701

[25] Kovalchin J , Krieger J , Genova M , et al. Macrophage‐specific chemokines induced via innate immunity by amino acid copolymers and their role in EAE. PLoS One. 2011; 6: e26274. 22194778 10.1371/journal.pone.0026274 PMC3240613

[26] Weber MS , Prod'homme T , Youssef S , et al. Type II monocytes modulate T cell‐mediated central nervous system autoimmune disease. Nat Med. 2007; 13: 935–43. 17676050 10.1038/nm1620

[27] Weber MS , Starck M , Wagenpfeil S , Meinl E , Hohlfeld R , Farina C . Multiple sclerosis: glatiramer acetate inhibits monocyte reactivity *in vitro* and *in vivo* . Brain. 2004; 127: 1370–8. 15090474 10.1093/brain/awh163

[28] Kala M , Rhodes SN , Piao WH , Shi FD , Campagnolo DI , Vollmer TL . B cells from glatiramer acetate‐treated mice suppress experimental autoimmune encephalomyelitis. Exp Neurol. 2010; 221: 136–45. 19879259 10.1016/j.expneurol.2009.10.015

[29] Aharoni R , Kayhan B , Eilam R , Sela M , Arnon R . Glatiramer acetate‐specific T cells in the brain express T helper 2/3 cytokines and brain‐derived neurotrophic factor *in situ* . Proc Natl Acad Sci U S A. 2003; 100: 14157–62. 14614135 10.1073/pnas.2336171100 PMC283562

[30] Goodin DS , Frohman EM , Hurwitz B , et al. Neutralizing antibodies to interferon beta: assessment of their clinical and radiographic impact: an evidence report: report of the Therapeutics and Technology Assessment Subcommittee of the American Academy of Neurology. Neurology. 2007; 68: 977–84. 17389300 10.1212/01.wnl.0000258545.73854.cf

[31] Conner J . Glatiramer acetate and therapeutic peptide vaccines for multiple sclerosis. J Autoimmun Cell Responses. 2014; 1: 1–11.

[32] Teitelbaum D , Brenner T , Abramsky O , Aharoni R , Sela M , Arnon R . Antibodies to glatiramer acetate do not interfere with its biological functions and therapeutic efficacy. Mult Scler. 2003; 9: 592–9. 14664472 10.1191/1352458503ms963oa

